# O11 Primary Sjögren’s syndrome vs MS: overlap or misdiagnosis?

**DOI:** 10.1093/rap/rkaa053.010

**Published:** 2020-11-03

**Authors:** Alaeldin Mohamednour, Maumer Durrani

**Affiliations:** Leicester Royal infirmary, Leicester, United Kingdom

## Abstract

**Case report - Introduction:**

Primary Sjögren’s syndrome (PSS) is a systemic autoimmune disease that mainly affects exocrine glands. Central nervous system (CNS) involvement in primary SS is extremely rare. In 10–20% of patients diagnosed with PSS, there are lesions in the central nervous system analogous to those presented in multiple sclerosis. We report a case of a 58-year-old female, diagnosed as PSS and multiple sclerosis (MS) (2007), but later, all neurological manifestations turned out to be related to PSS rather MS. This case illustrates how difficult it could be, distinguishing Sjögren’s with CNS involvement from MS, even to an expert clinician.

**Case report - Case description:**

A 58-year-old lady presented to Rheumatology clinic in 2010 with polyarthralgia, sicca symptoms and Raynaud’s. Immunology tests (positive anti- RO & anti-LA antibodies) and lymph node biopsy were highly suggestive of primary Sjögren’s. She was commenced initially on HCQ and prednisolone. Then Methotrexate was added in because she continued to struggle with inflammatory arthritis.

Her Sicca symptoms got gradually worse despite being on Acetylcysteine, Hylo Forte, cyclosporine and Dexamethasone eye drop. Therefore, autologous serum eye drops were tried with good response.

Her past medical history included Hypertension and knee OA. She has been under Neurology since 2007 for MS. Her original neurological symptoms were imbalance, dizziness, headaches, and tremor of the right arm which seem to be persistent with no definite relapses. MRI brain and spine were reported as normal with a few non-specific white matter areas, but the lumbar puncture result was positive for unmatched bands in the CSF.

Clinical examination revealed action tremor in the right upper limb. She had diminished vibration, pinprick, and cold temperature perception in a stocking distribution.

**Investigations:**

WBC 2.0, lymphocyte 0.62, DsDNA 1, C3 0.061, C4 0.01. CRP <5, PV 1.63, APS screen was negative

NCS: evidence of sensory and axonal neuropathy predominantly affecting lower limbs. CTCAP 2018 – showed calcification of parotid. No evidence of lymphoproliferative disorder.

The latest MRI 2019 showed two new lesions (right corpus &right striatum lesion) which according to Neuro-radiology MDT discussion were not typical of MS and more likely related to underlying CTD.

Based on these MRI findings and the recent history of skin vasculitis, the deterioration in her neurological condition was put down to primary Sjögren’s. Therefore, her treatment was escalated to cyclophosphamide during the COVID-19 pandemic with a particularly good outcome. She was then switched to MMF and her condition remained stable.

**Case report - Discussion:**

Neurological disorders are one of the rare manifestations of primary Sjögren’s. The first reports regarding the involvement of the nervous system in PSS were published in 1980. Distinguishing between multiple sclerosis and CNS-SS is not easy.

Not only because of similarities of the MRI findings, but also the course of the disease can be like MS, either chronic or relapsing and remitting. This usually leads to missing or delaying in the diagnosis as shown in this case.

However, Peripheral neuropathy is far much common in PSS rather MS which can help in differentiating these two conditions. Distal axonal sensory polyneuropathy is the most usual form of neuropathy in PSS as illustrated in this case. Furthermore, up to 75% of patients with SS and active CNS disease have been shown to have concomitant active peripheral vasculitis affecting the skin, muscles, and nerves. Our patient later developed skin vasculitis and peripheral neuropathy which made us think that all the neurological findings including the lesions on the brain are more likely to be related to PSS rather MS.

Cognitive disorders are common manifestations of CNS-SS such as attention disorder and memory deficit. Dementia-related to CNS-SS seems to be reversible after immunosuppressive treatment. A second MDT discussion took place and after considering the risk-benefit ratio, the decision was made to give cyclophosphamide. Patient was given all the information to make an informed decision. Patient asked for more time to think and discuss with her partner, but eventually, she had decided to have cyclophosphamide despite all the risks and uncertainties around the COVID-19 pandemic. Our patient has noticed significant improvement regarding cognition after completing cyclophosphamide treatment and she was pleased with this outcome.

**Case report - Key learning points:**

1/ Distinguishing between multiple sclerosis and CNS-SS is difficult

2/ neurophysiological tests should be considered even in asymptomatic patients as they contribute to the detection of early and subtle damage to the nervous system.

3/ Successful outcome being achieved with intensive immunosuppression despite all the uncertainties around the COVID-19 -19 pandemic.

4/ This case highlights the importance of communication and openness in shared decisions, especially while confronting uncertainties such as in COVID-19 pandemic.

